# Delineating novel diagnostic biomarkers and therapeutic targets for oral submucosal fibrosis: an integrative multi-omics and machine learning approach

**DOI:** 10.3389/fbinf.2026.1803111

**Published:** 2026-05-04

**Authors:** Chinmay Nitin Mokal, Piyush Agrawal

**Affiliations:** 1 Department of Oral and Maxillofacial Surgery, SRM Kattankulathur Dental College and Hospital, Chennai, India; 2 Division of Medical Research, SRM Medical College Hospital and Research Centre, Faculty of Medicine and Health Sciences, SRM Institute of Science and Technology, Chengalpattu, India

**Keywords:** areca nut, epithelial-mesenchymal transition, extracellular matrix remodeling, machine learning, multi-omics data, oral squamous cell carcinoma, oral submucous fibrosis

## Abstract

**Background:**

Oral submucosal fibrosis (OSF) is a chronic and progressive disorder, caused by chewing areca nuts, affecting the oral cavity and oropharynx. OSF is characterized by severe symptoms like severe burning sensation, restricted mouth opening, etc. Given the multifactorial and poorly understood molecular basis of the disease, there is a need for novel biomarkers and therapeutic targets.

**Method:**

We downloaded 3 RNA-seq, two microarray, one epigenomic, and one single-cell RNA-seq datasets from the gene expression omnibus database. Differentially expressed genes (DEGs) were characterized using DESeq2. Several analyses, including gene enrichment, immune cell infiltration, protein-protein interaction, and more, were performed. Machine learning models were developed using all DEGs and top5 selected features with leave one out cross validation technique. Independent validations were performed using two microarray datasets with appropriate statistical measures. Epigenetic analysis revealed hyper- and hypomethylated genes based on delta-beta values, and an integrative analysis of the transcriptome and methylome was performed to obtain key biomarkers. Single-cell analysis was performed to identify key cell types showing higher DEG expression.

**Result:**

DESeq2 analysis identified 29 upregulated and 15 downregulated DEGs. Upregulated DEGs show enrichment for the inflammatory, metabolic, and signaling processes, whereas downregulated DEGs were largely associated with metabolic processes. Immune cell enrichment analysis using CIBERSORTx shows higher enrichment of “T cells,” “mast cells,” and “macrophages” in OSF patients. We validated our findings in two independent microarray datasets and observed a similar gene expression pattern of DEGs. Machine learning performed using top5 features where Random Forest model achieved AUROC of 0.99 and AUPRC of 0.99. Further, ROC analysis and AUC plot show that DEGs can discriminate OSF patients from the normal population with high AUROC. Integrative analysis of methylation and transcriptomic data identified 11 genes as potential diagnostic biomarkers and therapeutic targets. Finally, single-cell analysis elucidates the higher expression of DEGs in “keratinocyte”, “epithelial cells” and “dendritic cells”.

**Conclusion:**

Integrative analysis identified 11 gene signatures as potential early diagnostic biomarkers and therapeutic targets for the OSF. Furthermore, the study hints towards mechanistic insight into potential mechanism leading to oral cancer. All the codes and ML models are provided at our GitHub repository https://github.com/agrawalpiyush-srm/OSF.

## 1 Background

Oral submucosal fibrosis (OSF) is one of the top oral malignant disorders and is chronic, progressive, and irreversible in nature. The disease is caused by chewing areca nut and using betel quid and predominantly affects the oral cavity and oropharynx. The patient suffers symptoms such as difficulty in mouth opening, burning sensation, dysplasia, and occasional facial deformity, ultimately affecting the patient’s quality of life (Tang et al., 2025). As per the World Health Organization (WHO) report, OSF is listed as one of the key oral malignant disorders, where around 3%–10% of OSF patients eventually develop oral squamous cell carcinoma (OSCC) (Warnakulasuriya et al., 2021; Warnakulasuriya, and Chen, 2022). Clinically, OSF is usually diagnosed at an advanced stage because of its slow and subtle progression. However, despite significant progress in understanding the pathobiology of several organ fibrosis (liver, lung, etc.), understanding of OSF is limited because of a lack of attention to this disease and the shortcomings in the field of research (Ray et al., 2016).

Epidemiological reports suggest that OSF predominantly affects individuals between the ages of 20 and 40 years of life. While it was primarily reported to be prevalent in males, recent reports suggest considerable geographic variation, where females have shown higher cases than males (Wang M et al., 2024). The common affected site includes buccal mucosa and retromolar trigone corresponding to habitual quid placement. OSF is highly prevalent in countries such as India, Sri Lanka, Vietnam, China, Myanmar, Taiwan, Bangladesh, and many other Pacific islands. Also, countries such as South Africa and the United Kingdom have shown the similar trend due to immigration of South Asian populations (Wollina et al., 2015).

OSF pathogenesis is a complicated process involving several factors, with arecoline, the principal alkaloid of areca nut, playing a central role. Arecoline stimulates fibroblasts to upregulate several growth factors and cytokines, including tumor necrosis factor alpha (TNF-α), basic fibroblast growth factor (bFGF), connective tissue growth factor (CTGF), transforming growth factor beta (TGF-β), alpha smooth muscle actin (α-SMA), and tissue inhibitor of metalloproteinases (TIMPs), while suppressing matrix metalloproteinases (MMPs) (Ullah et al., 2015). This imbalance leads to degradation of collagen and promotes excessive deposition of extracellular matrix (ECM), a hallmark of OSF. Additionally, arecoline-induced inflammation and increased reactive oxygen species (ROS) promote endothelial damage, reduced vascularity, thinning of epithelial tissue, and DNA damage. In addition, the high amount of copper present in commercial areca products leads to increased cross-linking of collagen, further enhancing tissue stiffness and restricted movement of the jaw (trismus) (Shen et al., 2020).

Epithelial-mesenchymal transition (EMT) plays a pivotal role in the development and progression from fibrosis to dysplasia and malignancy. Some of the key mediators regulating this transition include Snail-1, IL-6, YAP-1, vimentin, N-cadherin, BMP-4, and SMAD2/3, all of which lead to loss of epithelial integrity and acquiring invasive properties (Lamouille et al., 2014; Gonzalez, and Medici, 2014). Likewise, tumor suppressor genes (TSGs) such as KLF-6 (downregulated) and proteoglycans such as Testican-1 (upregulated) together drive EMT and remodeling of the ECM (Narla et al., 2001). In addition, senescent fibroblasts induced by arecoline via cytokines such as IL-6, IL-8, and GRO-α further enhance EMT and tumorigenic signaling (Ortiz-Montero et al., 2017). Several interconnected pathways comprising TGF-β, hypoxia, Wnt, MAPK/ERK, Akt/mTOR, and matrix-associated are involved in malignant transformation (Glaviano et al., 2023).

The current treatment focuses primarily on areca nut use cessation. Intralesional corticosteroids and hyaluronidase are used to treat mild to moderate cases, though responses vary (Warnakulasuriya, and Kerr, 2016). Curcumin has been used as a potential drug; however, it has shown limited benefit (Al-Maweri, 2019). Surgical excision of the fibrotic band has been performed in severe cases, and reconstruction was done using buccal fat pads, skin grafts, or laser excision techniques (Surej Kumar et al., 2010). However, till now, no therapy addresses the molecular drivers of the OSF, highlighting the requirement for mechanism-based approaches. In this study, we have analyzed several transcriptomic datasets (RNA-seq), methylation datasets, and single-cell datasets and further validated our findings on microarray transcriptomic datasets to characterize novel diagnostic and prognostic markers. Our analyses elucidated several key targets that can be used for therapeutic purposes.

## 2 Methodology

### 2.1 Dataset creation and processing

We searched and downloaded all the datasets associated with oral submucosal fibrosis (OSF) for the analysis from the gene expression omnibus (GEO) database. In total, five transcriptomic datasets, one epigenomic dataset, and one single-cell dataset were downloaded. Out of six datasets, two datasets are generated using microarray and three are generated using RNA-seq technologies. Details of these datasets are provided in Table 1. In the case of RNA-seq datasets, raw sequencing reads were downloaded and processed for further analyses. However, for the microarray dataset, GSE20170, we directly downloaded the normalized log2 ratio (Cy5/Cy3) for the analyses, whereas for GSE64216, raw data for the OSF patient and normal individual were downloaded and processed. In the case of the epigenomic dataset, GSE156669, the processed data was downloaded, and the list of significant differentially methylated genes (DMGs) was obtained. In the case of the epigenetic dataset, a p-value of ≤0.05 was used as a reference for statistical significance, as the adjusted p-value (FDR) provided a very small or negligible list of significant genes. For single-cell analysis, we found one dataset, GSE220978, which provided data for four patients. The dataset was processed using the Seurat 5.0 pipeline (Hao et al., 2021), and cell types were annotated using the ‘SingleR’ package (Aran et al., 2019).

**TABLE 1:** Dataset description. The table provides details of all the datasets used in the study.

Id	Type	Site	Sample size	References
GSE125866	RNA-seq	Oral tissue	Normal: 2 Case: 8	Zhou et al., 2019; Zhou et al., 2021
GSE274202	RNA-seq	Oral tissue	Normal: 2 Case: 2	Shetty et al. (2025)
GSE274203	RNA-seq	Oral tissue	Normal: 2 Case: 2	Shetty et al. (2025)
GSE20170	Microarray	Oral tissue for patient and Gingival tissue for normal	Normal: 10 Case: 10	Khan et al. (2011)
GSE64216	Microarray	Oral tissue	Normal: 2 Case: 4 (2 with dysplasia and 2 without dysplasia)	Not available
GSE156669	DNA methylation	Oral tissue for patient and buccal mucosa for normal	Normal: 5 Case: 7	Kundu et al. (2022)
GSE220978	10X Visium spatial single cell RNA-seq	Oral tissue	Case: 4	Zhi et al. (2024)

RNA-seq datasets were used for characterizing differentially expressed genes (DEGs), whereas microarray datasets were used for validation. Epigenomic and single-cell analysis datasets were further integrated and analyzed with RNA-seq datasets to obtain robust biomarkers driving OSF pathogenesis and can be used as potential therapeutic targets.

### 2.2 Differentially expressed genes (DEGs) characterization using RNA-seq dataset

For RNA-seq datasets, DEGs were characterized using DESeq2 software (Love et al., 2014) implemented in the R package. DESeq2 uses raw reads and implements negative binomial distribution to compute statistically significant DEGs. DESeq2 was implemented on individual datasets using default parameters to obtain the list of up- and downregulated DEGs. In case of GSE125866, all the genes with a LogFC ≥1 and adjusted p-value (FDR) ≤ 0.05 were called “upregulated,” and the genes with a LogFC ≤ -1 and adjusted p-value (FDR) ≤ 0.05 were called “downregulated”, whereas, in case of datasets GSE274202 and GSE274203, all the genes with a LogFC ≥1 and p-value ≤0.05 were called “upregulated,” and the genes with a LogFC ≤ -1 and p-value ≤0.05 were called “downregulated. The relaxed criteria for the above two datasets were used because of the lower sample size in these two datasets. Next, we combined all the DEGs (up and down) and computed the frequency of the unique genes. All the genes with frequency two or higher (present in at least two datasets) were further used for the downstream analyses. Also, the LogFC values of the DEGs were plotted in the form of a volcano plot using the “ggplot2” R package (Wickham et al., 2025).

Due to extremely small sample size in few datasets (n = 2 per group), we implemented bootstrapping-based DEG stability framework for effectively evaluating DEGs stability and mitigating noise arising from low sample size. For each RNA-seq dataset, we performed 500 bootstrap resampling iterations within case and control groups, followed by differential expression analysis in each iteration. We computed stability score for each gene as the proportion of iterations in which it was identified as significantly differentially expressed. Only genes exceeding a predefined stability threshold of 0.6 (value ranges b/w 0–1) were retained for downstream integration.

### 2.3 Gene enrichment analysis

We performed several gene enrichment analyses using the list of DEGs (up and down) and computed the enriched biological processes (BP), molecular functions (MF), and cellular compartments (CC) using the Enrichr database (Kuleshov et al., 2016). We also looked for the enriched KEGG pathways and the disease terms associated with the DEGs using the Enrichr database. The top 20 statistically significant enriched terms (if present) were shown in the form of a dotplot using the “ggplot2” R package. In the dotplot, “gene size” represents the number of DEGs present in the enriched term; “gene ratio” shows the fraction of the DEGs present among the total genes present in the term, and -log10(P-value) shows if the gene is statistically significant or not.

### 2.4 Protein-protein interaction (PPI) network using STRING

Protein-protein interaction (PPI) analysis was performed using the STRING database (Szklarczyk et al., 2019). A list of the DEGs was provided as an input, and ‘*homo sapiens*’ was selected as the ‘Organism’ against which the network was created. The network was further refined based on the “minimum required interaction score,” with the score of 0.7 to obtain the network where the proteins are interacting with high confidence. High-resolution images of PPI networks and the enriched terms associated with them were downloaded as a form of STRING output.

### 2.5 Gene set variation analysis

Gene Set Variation Analysis (GSVA) (Hänzelmann et al., 2013) was performed on variance-stabilized expression data obtained from DESeq2 to assess pathway-level functional alterations. The variance stabilizing transformation (VST) technique (Lin et al., 2008) was implemented for normalizing the raw data and ensuring comparability across samples. For hallmark gene sets, we used the Molecular Signatures Database (MSigDB) (Liberzon et al., 2015) as reference pathways. Next, to estimate sample-wise pathway activity scores, GSVA was applied, enabling the evaluation of coordinated biological processes. As the sample size was smaller, a non-parametric, trend-based approach was implemented, wherein pathway activity differences between the two groups (treated and control) were assessed by comparing group-wise mean GSVA scores rather than depending on formal statistical testing. This approach facilitated robust identification of consistent pathway-level trends while avoiding overestimation of significance in small cohorts.

### 2.6 Immune cell infiltration analysis

CIBERSORTx (Steen et al., 2020) and xCell 2.0 (Angel et al., 2025) were used to estimate the enrichment of different immune cell types in the OSF patients and the healthy control. CIBERSORTx is a deconvolution tool that implements a machine learning algorithm and analyzes gene expression data to estimate the proportion and type of cells in a mixed sample. In contrast, xCell 2.0 uses the estimation of the enrichment score with spillover compensation for cell types that are highly related to each other. Here, in this case, we used the data from GSE125866, as the two other RNA-seq datasets (GSE274202 and GSE274203) have very few samples (2 patients and two controls), which may be insufficient to provide meaningful statistical inference and can introduce bias or unstable estimates in cell-type proportion analysis. The data were normalized in the form of log2 (TPM+1) gene expression, and the resulting matrix was uploaded to the website for the analysis. In the case of CIBERSORTx, we used the default ‘LM22’ gene signature, and ‘100’ was used as permutations for significance. The output was downloaded in the form of ‘pdf’ and ‘csv’ files, providing the enrichment of 22 cell types in OSF patients and normal patients. We further computed the Spearman’s correlation among the 22 different immune cell types with each other. For this correlation analysis, we used only the estimate of OSF patient data. This approach has already been used in previous analyses (Mitra et al., 2025). However, in case of xCell, we used the default parameters. The output file provided enrichment of 64 immune and stromal cell types. For visualization, we normalized the xCell output score using the z-score technique and a heatmap was created.

### 2.7 Machine learning model development and feature selection

We developed five different machine learning [ML] models using the Python package scikit-learn (Pedregosa FABIANPEDREGOSA et al., 2011) using the three different RNA-seq datasets combined into one. We developed Support Vector Machine (SVM), Random Forest (RF), AdaBoost, GradientBoost, and ExtraTree classifiers, which can classify OSF patients from normal. We performed the leave-one-out cross-validation (LOOCV) technique due to the limited sample size. In this technique, one sample is used for testing while the model is trained on the remaining samples. This process is repeated until all samples get used once for testing. We performed 20 such iterations to optimize hyperparameters within each training set using internal cross-validation to prevent information leakage. Post all iterations, model performance was aggregated, and performance was computed using the area under the receiver operating characteristic curve (AUROC) and the area under the precision–recall curve (AUPRC). As all the features do not add value to the model performance, we performed feature selection using the recursive feature elimination (RFE) technique (Guyon et al., 2002). We identified the top five features/genes contributing to model-specific estimators. Post characterization, we again implemented LOOCV just like above and reported the performance.

We further employed a nested cross-validation strategy, to obtain an unbiased model performance estimate and address the risk of overfitting due to limited sample size. Particularly, an outer stratified k-fold cross-validation (k = 3) was used to evaluate model performance, while for hyperparameter tuning, inner stratified k-fold cross-validation (k = 3) was used. This strategy ensures that model selection and evaluation are performed on independent data splits. AUROC was used for assessing the model performance and the results were reported in terms of mean and standard deviation across outer folds. To further ensure the model performance statistical significance, permutation testing was performed by randomly shuffling class labels 1000 times (n = 1000 iterations) and repeating the nested cross-validation procedure. At the end of this permutation testing, p-value was reported, which is computed as the proportion of permuted datasets achieving performance equal to or greater than that of the original model.

### 2.8 External validation in independent datasets

Validation of DEGs was performed using two transcriptomic datasets, GSE20170 and GSE64216, generated using microarray technologies. In the case of GSE20170, the normalized log2 ratio (Cy5/Cy3) was used for the analyses, whereas for GSE64216, raw data for the OSF patient and normal individual were downloaded and processed for the analyses. Also, in the case of GSE64216, we had two types of OSF patients: one with dysplasia and one without dysplasia. We compared the gene expression pattern and the directionality to see if upregulated DEGs are significantly expressed higher in OSF patients compared to the normal population and *vice versa*. We also validated our gene signatures by performing plotting receiver operating characteristic (ROC) curve and area under curve (AUC) to see if our potential DEGs can classify OSF patients with normal using their gene expression values. To keep the robustness and statistical significance intact, we used the gene expression from the microarray dataset GSE64216. We also plotted the directionality of the gene on the AUROC curve. The ‘pROC’ R package (Robin et al., 2025) was used for the AUROC plots.

### 2.9 Epigenomic dataset processing and analysis

We downloaded the dataset by Kundu et al. (2022), where genome-wide DNA methylation changes in OSF patients were performed. We downloaded the processed data where the information of each probe, gene(s) corresponding to the probe, mean beta methylation values in OSF and normal, and p-value and adjusted p-value was provided. As the number of significant probes as per adjusted p-value was very low, we used p-value and obtained the list of significant differentially methylated genes. Genes with positive delta beta values (i.e., mean value in OSF -mean value in normal) were called hypermethylated genes, whereas genes with negative delta beta values were termed hypomethylated genes.

### 2.10 Single cell data processing and analysis

We were able to obtain one single-cell dataset by Zhi et al. (2024), where they performed spatial transcriptomic and metabolic analyses for the OSF-derived oral squamous cell carcinoma (OSCC) and its tumor microenvironment (TME) in four patients. We used this data to perform single-cell analyses and observe the expression of our DEGs in different cell types. We downloaded the three key files required for the analyses, i.e., the barcode, the matrix, and the feature file, and processed them from scratch. We used the Seurat 5.0 pipeline to process the single data and perform the quality control before analysis. Quality control was performed by analysing the distribution of detected features (nFeature_RNA), total counts (nCount_RNA), and mitochondrial percentage. As it is spatial data, low-quality spots with less than 200 features and high mitochondrial percentage (>10%) were removed based on their distribution. We did not keep any maximum number of features cutoff, which is applicable to a multi-cellular nature of spatial transcriptomics spots. This ensures that the high transcript density resulting from multiple cells, tumor microenvironments, or mixed cell types did not get filtered out. Next, we performed basic analysis such as dimensionality reduction using principal component analysis (PCA) followed by uniform manifold approximation and projection (UMAP), finding neighbors and clusters. After this, we annotated the cell type using the “SingleR” (Aran et al., 2019) library and its default parameters and the Human Primary Cell Atlas (HPCA) as a reference to find the enriched cell type in each of the four patients.

Gene expression z-scoring was performed for all genes across all cell types. For each cell type, a mean z-score was obtained per gene, and all the genes with a positive z-score were considered as expressed in that cell type. Next, we computed the Log2 (Obs/Exp) fraction of genes expressed in each cell type. Here ‘Obs’ is the number of genes with positive z-scores from our set of DEGs, and ‘Exp’ is all the genes with positive z-scores. The resulting values were further normalized between −1 and one for better visualization. This is a well-established methodology and has been implemented in our previous work (Agrawal et al., 2024; Patiyal and Agrawal, 2025).

## 3 Results

### 3.1 Summary of the study

In this study, we identified DEGs (up- and downregulated) using three different RNA-seq datasets using a frequency-based approach. We performed various analyses using these DEGs, such as gene enrichment analyses, KEGG pathway and disease term enrichment analysis using Enrichr, PPI analysis using STRING, and immune cell infiltration analysis using CIBERSORTx. Next, we validated these genes in two independent datasets generated using microarray technologies, where we compared the gene expression pattern in OSF patients and the normal population and performed ROC analysis to see if these genes can classify OSF patients from normal with high accuracy. Afterwards, we analyzed methylation data to identify differentially methylated genes and their overlap with our DEGs. Next, we analyzed single-cell data to find the cell types in which DEGs were expressed. We integrated transcriptomic and epigenomic data to find common, robust diagnostic markers against OSF. Lastly, we selected the key therapeutic targets and performed virtual screening and docking to identify potential drugs. We identified 29 genes as upregulated and 15 as downregulated in 2/3 datasets. Gene enrichment analysis identified the key biological processes, molecular functions, and cellular compartments associated with OSF. Also, the enriched pathways and disease terms were found to be associated with OSF. PPI analysis interacting partners only for the upregulated genes, which were enriched for OSF and OSCC-related terms. Immune cell infiltration analysis revealed enrichment of key immune cell types in OSF patients compared to normal. External validation of the DEGs in microarray datasets further strengthens our findings, as they can clearly show the significant change in gene expression pattern in OSF compared to normal. The ROC curve shows that DEGs can classify OSF and normal with AUC up to 0.99. Epigenetic analysis provided several hypo- and hypermethylated genes, and several of them were found to be common with DEGs. Single-cell analysis revealed key cell types where the DEGs were found to be highly expressed. Lastly, drug repurposing against key targets that were upregulated and hypomethylated in OSF patients revealed putative drugs for therapeutic use.

### 3.2 Differentially expressed genes are enriched for key biological processes

**FIGURE 1:**
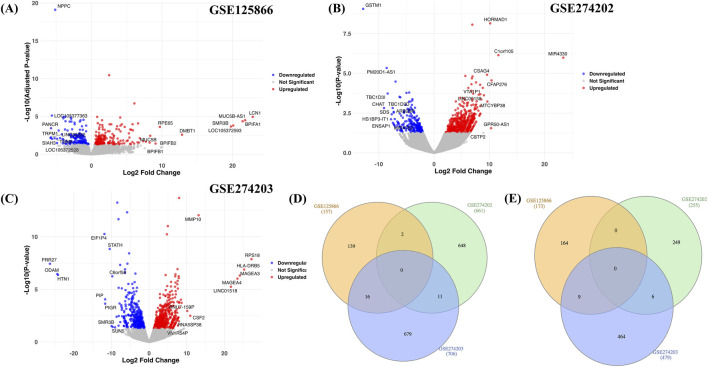
Summary of RNA-seq datasets. The violin plot displays the DEGs for three RNA-seq datasets **(A–C)**, along with their log2 fold change values on the x-axis and -log10 (adjusted p-value/p-value) values on the y-axis. Panel **(D)** shows the commonality among the upregulated DEGs obtained in each of the three datasets, and Panel **(E)** shows the commonality among the downregulated DEGs obtained in each of the three datasets.

DESeq2 was implemented, and statistically significant DEGs (up- and downregulated) were obtained from all three RNA-seq datasets (Supplementary Tables S1–S3). Any gene with LogFC ≥1 and FDR ≤0.05 was called upregulated, and LogFC ≤ -1 and FDR ≤0.05 was called downregulated. In the case of GSE125866, in total 157 upregulated and 173 downregulated DEGs were obtained; for GSE274202, 661 up and 255 downregulated genes were obtained, and in the case of GSE274203, 706 up and 479 downregulated genes were reported (Supplementary Table S4). Violin plots showing the LogFC distribution for all three datasets are shown in Figures 1A–C. Next, we compiled the genesets of up- and downregulated DEGs and computed the frequency and selected those genes that were present in at least two datasets. In total, we obtained 29 upregulated and 15 downregulated genes (Supplementary Table S5), which were used for further downstream analyses (Figures 1D,E).

As the sample sizes of few datasets are limited, we further evaluated the robustness of the characterized differentially expressed genes (DEGs) by performing a bootstrap-based stability analysis. Across 500 resampling iterations, 489, 4382, and 1094 genes were identified as stable (stability score ≥0.6) in GSE125866, GSE274202, and GSE274203, respectively (Supplementary Table S6). This observation indicates variability in transcriptomic consistency across three datasets.

First, we compared the DEGs overlap in a dataset-specific manner. In the case of GSE125866, 64.8% of genes were common (214/330), i.e. 214 genes out of 489 were in common with 330 DEGs initially obtained. Likewise, in the case of GSE274202 and GSE274203, 12.8% (117/916) and 42.7% (506/1185) genes were common. In addition, we also compared the overlap with 44 DEGs obtained via a frequency-based approach with 439, 4382, and 1094 genes (stable genes) and observed overlap of 61.4% (27/44), 29.5% (13/44), and 38.6% (17/44) in GSE125866, GSE274202, and GSE274203, respectively. Overall, GSE125866 showed strong stability, and GSE274203 showed moderate stability, whereas GSE274202 showed lower stability, suggesting higher sensitivity to sampling variability.

In summary, the results highlight that although the degree of stability varies across datasets, a substantial proportion of both dataset-specific and frequency-derived DEGs remain reproducible under perturbation. This shows the effectiveness of the bootstrapping approach in classifying robust transcriptional signals from potential noise, thereby strengthening the reliability of downstream integrative analyses. Stability scores for all the genes for each dataset are provided in Supplementary Table S7.

**FIGURE 2:**
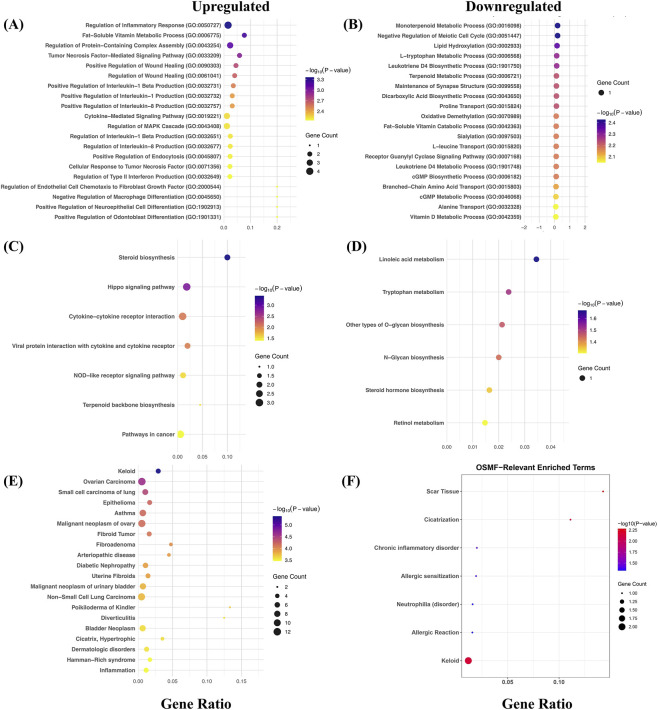
DEGs are enriched for key processes, pathways, and diseases. Panel **(A,B)** represent the top 20 enriched biological processes associated with up- and downregulated DEGs, respectively. Panel **(C,D)** represent the top enriched KEGG pathways associated with up- and downregulated DEGs, respectively. Panel **(E,F)** represent the top enriched disease terms associated with up- and downregulated DEGs, respectively. The Enrichr web-based tool was used to compute these terms with default parameters.

Next, we looked at the enriched biological processes associated with these DEGs using the Enrichr tool. As shown in Figure 2A, upregulated DEGs show the enrichment of processes related to the immune system, signaling, and metabolic pathways, such as “Regulation of Inflammatory Response,” “Fat-Soluble Vitamin Metabolic Process,” “Tumor Necrosis Factor-Mediated Signaling Pathway,” “Regulation of MAPK Cascade,” and more. These terms have been shown previously in independent studies explaining the underlying mechanism in OSF pathogenesis. This can be explained by the fact that in OSF, chronic inflammation plays a crucial role in disease progression. Several reports have highlighted the inflammatory response and Tumor Necrosis Factor (TNF)-mediated signaling pathways as key processes. Rajalalitha and Vali, in a study, reported the elevated levels of TNF-α and IL-1β, promoting activation of fibroblasts and excessive collagen deposition, a key characteristic linking inflammation to fibrosis (Rajalalitha, and Vali, 2005). Similarly, Tilakaratne et al. discussed the role of MAPK pathways in influencing differentiation of myofibroblasts and extracellular matrix (ECM) remodeling in OSF, highlighting its role in fibrotic events (Tilakaratne et al., 2016). Likewise, downregulated genes were found to be associated with processes largely related to metabolism, such as “L-tryptophan Metabolic Process,” “Terpenoid Metabolic Process,” “Fat-Soluble Vitamin Catabolic Process,” etc. (Figure 2B). Previous studies have shown the correlation of downregulation of these processes with the OSF progression. For example, Angadi et al. reported that due to metabolic imbalance, antioxidant defense and epithelial integrity are affected, ultimately leading to fibrosis (Angadi, and Rekha, 2011).

Similar to BP, we also looked at the molecular functions (MF) and cellular compartments (CC) associated with the DEGs. In the case of MF, upregulated DEGs were enriched for terms such as “Cytokine Activity,” “Receptor Ligand Activity,” “MHC Protein Binding,” etc. (Supplementary Figure S1A), whereas downregulated DEGs were found to be associated with terms such as “Iron Ion Binding,” “C-X-C Chemokine Binding,” etc. (Supplementary Figure S1B). These terms have been previously reported as well and validate our observation (Haque et al., 1998; Peng et al., 2022; Yadahalli et al., 2024). Likewise, for CC, upregulated genes were found to be enriched for only two terms, “Serine Protease Inhibitor Complex” and “Nuclear Outer Membrane,” whereas downregulated genes were enriched for terms such as “Platelet Dense Granule Lumen,” “Golgi Stack,” etc. (Supplementary Figures S2A,B). Our finding was supported by previous studies as well (Ranjitham et al., 2023; Chakraborti et al., 2025). A complete list of all the BP, MF, and CC associated with DEGs (up and downregulated) is provided in the Supplementary Table S8–S10.

We further performed analysis to identify KEGG pathways and disease terms associated with DEGs, using the Enrichr database. Upregulated genes were enriched for pathways such as “Steroid biosynthesis,” “Hippo Signaling,” “Pathways in Cancer,” etc. (Figure 2C). Steroid biosynthesis is associated with the production of steroid hormones such as glucocorticoids, which normally inhibits collagen synthesis and TGF-β signaling. Any dysregulation in this pathway may lead to collagen accumulation and fibrotic events (El-Okdi et al., 2008). Likewise, Hippo signaling regulates the key processes such as cell division, apoptosis, *etc.*, via YAP/TAZ transcription co-activators. It has been observed in OSF patients that areca nut chewing activates YAP/TAZ, leading to myofibroblast differentiation and excessive ECM deposition (Kazemi et al., 2025). Likewise, downregulated genes were associated with metabolic pathways such as “Linoleic acid metabolism,” “N-Glycan biosynthesis,” etc. (Figure 2D). We also looked for the disease terms enriched for DEGs, and we found that upregulated DEGs were associated with terms such as “Keloid,” “Fibroid Tumor,” etc., which are directly associated with OSF (Figure 2E). For example, “keloid” is defined as an area of irregular tissue that is formed at the site of a scar or injury. Though it is mainly found in skin fibrosis, several reports of keloid have been reported in OSF (Reilly et al., 1980; Vannala et al., 2021). In the case of downregulated genes, we observed the association of disease terms such as “Keloid”, “Scar Tissues”, “Allergic Sensitization”, “Chronic Inflammatory disorder”, etc. (Figure 2F). These terms points towards shared molecular pathways rather than direct disease specificity. Importantly, terms related to fibrosis corresponds to excessive deposition of ECM, a characteristic feature of OSF, whereas inflammatory disease terms reflect chronic immune activation and cytokine dysregulation underlying disease progression.

A complete list of all the pathways and disease terms associated with DEGs is provided in the Supplementary Tables S11,S12.

### 3.3 PPI analysis reveals key interacting partners

**FIGURE 3:**
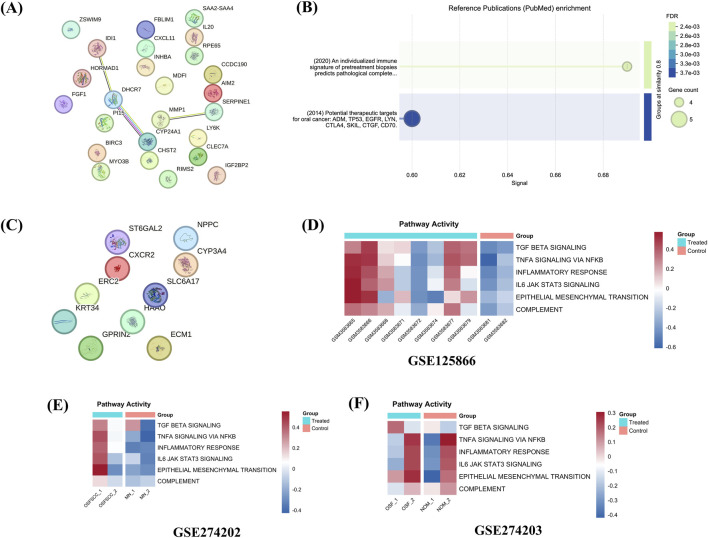
Protein-protein interaction and gene set variation analysis. Panel **(A)** shows the interaction among the upregulated DEGs; Panel **(B)** shows the enriched reference publication terms associated with upregulated DEGs, and Panel **(C)** shows the interaction among the downregulated DEGs. STRING database was used to perform this analysis. Panels **(D–F)** show the pathway activity score in the form of a heatmap of key pathways computed using GSVA analysis.

PPI analysis was performed using the STRING database, where a list of DEGs was provided as an input with *Homo sapiens* as the organism and 0.7 as the minimum required interaction score. In the case of upregulated proteins, we observed two interactions, one between proteins *IDI1, DHCR7,* and *CYP24A1* and the other between genes *MMP1* and *SERPINE1* (Figure 3A). Reference Publications (PubMed) enrichment analysis implemented in STRING shows two processes: (a) An individualized immune signature of pretreatment biopsies predicts pathological complete response to neoadjuvant chemoradiotherapy and outcomes in patients with esophageal squamous cell carcinoma and (b) Potential therapeutic targets for oral cancer: ADM, TP53, EGFR, LYN, CTLA4, SKIL, CTGF, and CD70 (Figure 3B). However, in the case of downregulated proteins, we did not find any interactions (Figure 3C).

We further complemented the PPI network analysis, which shows very sparse interactions, particularly for the downregulated DEGs, where no interaction was seen. As we do not know whether the observed interactions drive disease-relevant signaling, we performed pathway-level activity assessment using gene set variation analysis (GSVA), implemented in the R package, to evaluate the functional consequences of the identified gene interactions. Our analysis showed increased activity of key pathways associated with OSF pathogenesis, such as TGF-β signaling, TNFα signaling via NF-κB, IL6-JAK-STAT3 signaling, complement activation, and epithelial-mesenchymal transition in treated samples (Supplementary Table S13). We further plotted the activity of these OSF-relevant pathways across three datasets (Figures 3D–F) via heatmaps, which demonstrated a consistent pattern of pathway activation in OSF patients compared to controls. The above-mentioned pathways are well-known mediators of ECM remodeling, inflammatory responses, and fibroblast activation, thereby providing functional context to the observed PPI network. Therefore, by associating pathway activity with network topology and cross-dataset consistency, it supports the biological relevance of the reported DEGs and suggests that observed interactions are not purely descriptive associations; instead, they are coordinated and disease-associated signaling processes.

### 3.4 Immune cell infiltration analysis shows high infiltration of T cells (CD4 and CD8) and macrophages in OSF patients

**FIGURE 4:**
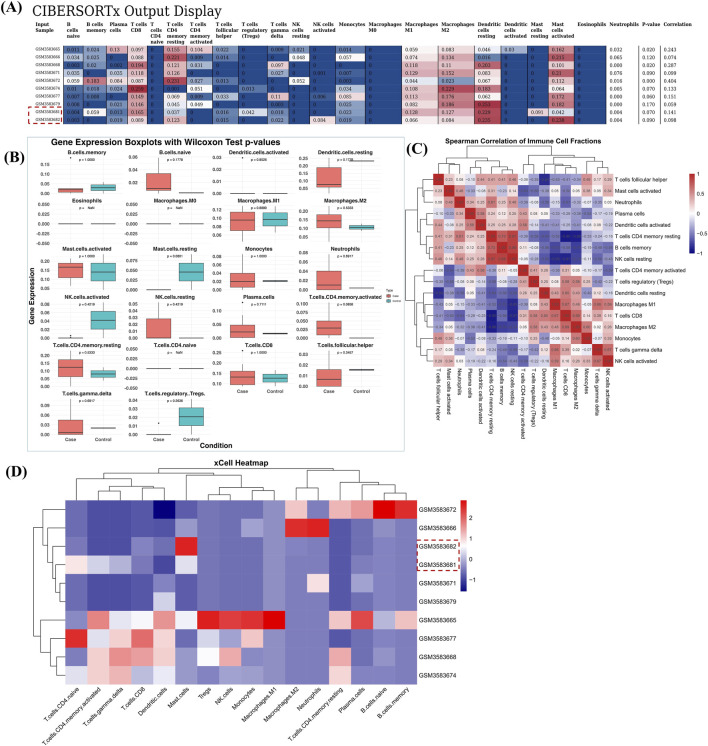
Immune cell enrichment analysis. Panel **(A)** shows the estimated proportions of 22 cell types, computed by CIBERSORTx, ranging in value between 0 and 1, p-value, and correlation for OSF patients and normal counterparts (highlighted by a red box). Panel **(B)** shows the statistical significance computed between cell types and their quantitative estimation in OSF patients in the form of a boxplot. The Mann-Whitney test was performed for computing the statistical significance. Panel **(C)** represents the heatmap of Spearman correlation computed among the various cell types enriched in OSF patients. Panel **(D)** shows the estimated proportions of various cell types computed by xCell, ranging in value between z-score of −2 and two for OSF patients and normal counterparts (highlighted by a red box).

Immune cell infiltration in OSF patients compared to control was computed by running two widely used packages CIBERSORTx and xCell on the GSE125866 dataset. As shown in Figure 4A, in case of CIBERSORTx, we observed high enrichment of “T cells CD4 memory resting cells” and “T cells CD8”, representing persistent antigenic stimulation and forming an immune microenvironment consistent with chronic inflammation and potential immune exhaustion (Farber et al., 2014; Wherry, and Kurachi, 2015). We also observed high infiltration of M1 and M2 macrophages in several OSF patients compared to normal. Increased infiltration of macrophages, particularly M2, highlights the chronic inflammation and fibrotic nature of the disease. M2 macrophages secrete cytokines that are pro-fibrotic in nature, such as IL-10, IL-13, and TGF-β, and are associated with collagen deposition and fibroblast activation, key characteristics of OSF (Wynn, and Vannella, 2016). Mast cells were found to be highly enriched in OSF patients as well, highlighting their potential role in the release of fibrogenic mediators, promoting the tissue stiffness and epithelial atrophy (Galli, and Tsai, 2008). In contrast, higher enrichment of dendritic cells and NK cells was reported in normal oral mucosa compared to OSF, highlighting the role of these cells in maintaining immune tolerance and tissue homeostasis. Statistical significance of enrichment of each of the 22 cell types was further computed using the Mann-Whitney statistical test and is shown in Figure 4B.

We further computed the correlation among the 22 immune cell types using Spearman correlation to observe distinct co-regulatory patterns among immune cell populations within the microenvironment (Figure 4C). We observed a high correlation of M2 macrophages with monocytes and T cells CD8 and of M1 macrophages with T cells CD8 and T cells gamma delta. The high correlation with monocytes indicates a coordinated activation of the innate immune system, highlighting their shared origin from myeloid cells and role in tissue remodeling and inflammation (Murray, and Wynn, 2011). Likewise, positive correlation with T cells (CD8 and gamma delta) shows the active crosstalk between macrophages and cytotoxic T cells, suggesting that cytokines derived from macrophages sustain cytotoxic T cell activation, while IFN-g and TNF-a derived from T cells drive macrophage polarization. The crosstalk between these two components collectively drives chronic inflammation and fibrosis. Conversely, there is a strong negative correlation of resting and memory B-cells, NK-cells, plasma cells, and subsets of T cells (T cells CD4) with cells such as monocytes, macrophages, and subsets of T cells (regulatory and CD4 activated), suggesting a reciprocal balance between the innate and adaptive immune systems, a well-established phenomenon in diseased conditions such as cancer and fibrosis (Mantovani et al., 2017). This inverse relationship between activated and resting cells also reflected the dynamic transition between the immune activation and resolution phases (Wang R et al., 2024). Overall, we observed a well-balanced functional immune plasticity during which the innate and adaptive immune systems shift and regulate the immunological tone of the tissue microenvironment.

In the case of xCell, similar enrichment of cell types was reported, as in CIBERSORTx, except for one or two differences. For example, xCell also reported high enrichment of macrophages, T cells CD4 memory resting cells, and T cells CD8. In addition, a few patients show higher enrichment of other cell types such as B cells and monocytes. However, in contrast to CIBERSORTx results, dendritic cells and NK cells were more enriched in OSF patients compared to control (Figure 4D). One possible reason for such a phenomenon might be attributed to the methodological variations in the application of the deconvolution strategy and the reference signature. CIBERSORTx uses constrained regression method to estimate the relative cell proportions, whereas xCell uses the estimation of the enrichment score with spillover compensation for cell types that are highly related to each other. Therefore, the observed differences may reflect the contextual representation of the immune compartment rather than actual biological inconsistency. Enrichment score of various cell types obtained by xCell is provided in Supplementary Table S14.

### 3.5 Machine learning models exhibits high potential in classifying OSF patients from normal

**FIGURE 5:**
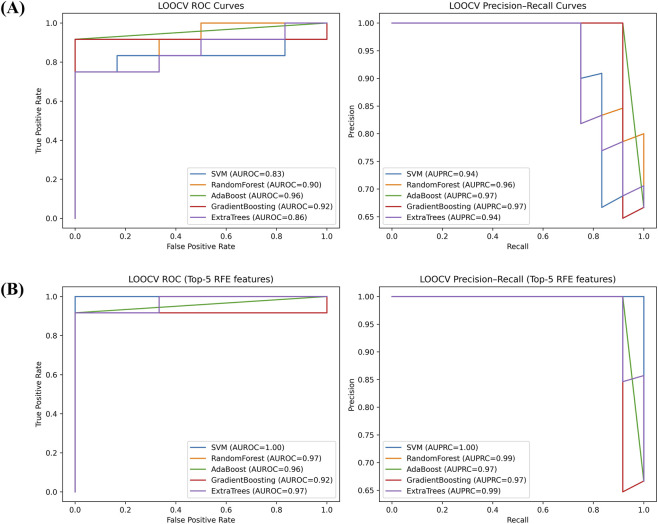
Machine learning performance. Panel **(A)** shows the AUROC and AUPRC values of five different ML classifiers developed using all DEGs; whereas Panel **(B)** shows the AUROC and AUPRC values of five different ML classifiers developed using top5 features characterized using recursive feature elimination (RFE) technique.

Leave one out cross validation technique was implemented on the training dataset, where we have in total 18 samples (12 positive and six negative) and 39 features (common DEGs in all three RNA-seq datasets). In total, 18 iterations were performed where 17 samples were used for training and one sample was used for testing. Performance on all the 18 iterations were aggregated and average AUROC and AUPRC was reported. As shown in Table 2 and Figure 5A, Adaboost model performed best among all the models and achieved the AUROC of 0.96 and AUPRC of 0.97 followed by GradientBoost model with AUROC of 0.92 and AUPRC of 0.97.

**TABLE 2:** Machine learning models performance. Performance of five different classifiers were evaluated on the training dataset using LOOCV technique. The model was evaluated using full features as well as using top5 features, selected through recursive feature elimination (RFE) technique. Best parameters were provided and the performance was reported in terms of AUROC and AUPRC.

	All features			Top5 features		
Machine learning classifier	Parameter	AUROC	AUPRC	Top5 features Parameter	AUROC	AUPRC
SVM	C: 1, gamma: 0.001; kernel: linear	0.84	0.94	CDC190; CXCR2; INHBA; MMP1; SAA2-SAA4	1	1
RF	max_depth: None; min_sample_leaf: 4; n_estimators: 500	0.90	0.95	IL20; RPE65; INHBA; MMP1; SAA2-SAA4	0.99	0.99
Adaboost	learning_rate: 0.01, n_estimators: 50	0.96	0.97	SERPINE1; RPE65; SLC6A17; ST6GAL2; ZSWIM9	0.96	0.97
GradientBoost	learning_rate: 0.05, max_depth: 2; n_estimators: 100	0.92	0.97	SERPINE1; RPE65; SLC6A17; ST6GAL2; ZSWIM9	0.92	0.97
ExtraTree	max_depth: 10; min_sample_leaf: 2; n_estimators: 200	0.86	0.94	IGF2BP2; IL20; INHBA; RPE65; SAA2-SAA4	0.97	0.99

We further performed feature selection using recursive feature elimination technique and reported top5 genes contributing to the individual model performance (Table 2). We observed different techniques characterized different features. For example, boosting algorithms (Adaboost and GradientBoost) identified same five features, whereas tree-based algorithms (Random Forest and ExtraTree) identified similar genes. We next used these top5 features for each classifier and performed LOOCV just like above and reported the performance again. We observed that post feature selection, performance of the models increased significantly, highlighting the biological relevance of the selected genes in classifying the OSF patients from normal. We observed that post feature selection, tree-based algorithms performed best compared to boosting-based classifiers. RF and ExtraTree classifiers achieved AUROC of 0.99 and 0.97 and AUPRC of 0.99 respectively (Table 2; Figure 5B).

However, due to fewer samples in test dataset (4 patient and two control), we could not evaluate the performance of the model on an independent dataset.

Considering the relatively small number of samples within the dataset, there is an inherent risk of overfitting, especially with respect to high-dimensional gene expression data. Conventional techniques including LOOCV has proven record of providing overly optimistic performance estimates under such conditions, especially when feature selection and model tuning are not adequately controlled.

Henceforth, to address these issues and provide a more comprehensive evaluation of the generalization capability of the models, we adopted the nested cross-validation strategy. Furthermore, we conducted 1000 times permutation testing (n = 1000) by randomly shuffling class labels to ensure that model performance was not by chance. As a result, among all machine learning models, Adaboost and GradientBoosting performed best with average AUROC of 0.96 ± 0.058 (Supplementary Table S15). Furthermore, the permutation testing achieved the p-value of 0.002, indicating that the model captures meaningful biological signal rather than noise.

Overall, this strategy not only provide a more conservative and statistically rigorous assessment of model performance acknowledging the limitations imposed by sample size.

### 3.6 Validation in external dataset strengthen the findings

**FIGURE 6:**
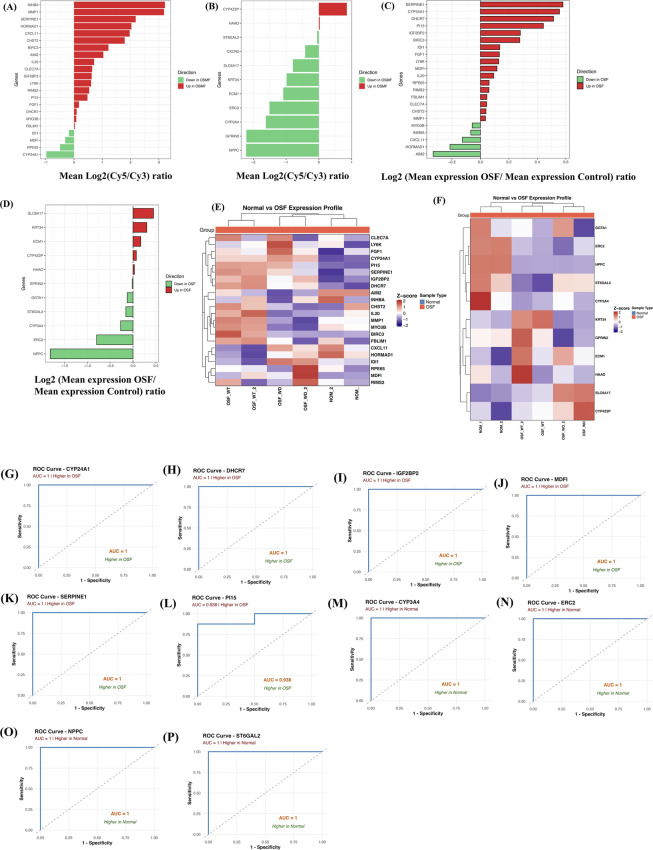
Validation of DEGs in external dataset. Panel **(A,B)** show the gene expression value (Log2 (Cy5/Cy3)) for the dataset GSE20170, whereas Panel **(C,D)** show the gene expression value (Log2 (Mean expression in OSF/Mean expression in normal)) for the dataset GSE64216. Red bars show higher expression in OSF patients and green bars in the normal population. Panel **(E,F)** show the gene expression pattern in OSF patients and the normal population for the upregulated and downregulated DEGs, respectively, in the form of a heatmap. Panel **(G–L)** shows the ROC analysis and AUC plot for the upregulated DEGs discriminating OSF patients from normal, whereas Panels **(M–P)** show the ROC analysis and AUC plot for the downregulated DEGs discriminating OSF patients from normal. Heatmap and ROC analyses were performed using the GSE64216 dataset.

We validated our DEGs in two external datasets obtained where transcriptome data of OSF patients and the normal population was generated using microarray technologies. In the case of 29 upregulated DEGs, data for only 22 genes were present, and for 15 downregulated DEGs, data for only 11 genes were present in the external validation dataset. We analyzed the gene expression pattern of our DEGs common with external datasets (22 up and 11 down) and observed that upregulated DEGs show higher gene expression in OSF patients, whereas downregulated DEGs show lower gene expression in OSF patients compared to normal counterparts. In the case of GSE20170, except for *IDI1, MDFI, RPE65,* and *CYP24A1,* all the upregulated DEGs show higher gene expression in OSF patients with log2 values ranging between 0 and 3.5 (Figure 6A), whereas in the case of downregulated DEGs, all the genes show lower gene expression in OSF patients with log2 values ranging between 0 and -2, except for genes *CYP4Z2P* and *HAAO* (Figure 6B). Similarly, in the case of GSE64216, upregulated DEGs show higher expression in OSF patients with a log2 range in between 0 and 1, except for genes *MYO3B, INHBA, CXCL11, HORMAD1,* and *AIM2,* which show opposite behavior (Figure 6C), whereas in the case of downregulated DEGs, all genes except *SLC6A17, KRT34, ECM1, CYP4Z2P*, and *HAAO* show lower gene expression in OSF patients with a log2 range in between 0 and -2 (Figure 6D).

We further plotted the gene expression of DEGs in the form of a heatmap by normalizing the gene expression and computing the z-score for better visualization in a sample-specific manner. We used dataset GSE64216, as sample-specific gene expression is provided for this dataset only and not for GSE20170. As shown in Figures 6E,F, it is clearly visible that our DEGs show a distinct gene expression pattern, which can be further used as potential diagnostic markers. We also observed a pattern within OSF patients with dysplasia and without dysplasia as well. For example, genes such as *RPE65, MDFI,* and *RIMS2* were highly expressed in OSF patients with dysplasia compared to OSF without dysplasia and the normal population. Likewise, genes such as *BIRC3, IL20,* and *MMP1* were expressed in OSF without dysplasia compared to OSF with dysplasia and the normal population (Figure 6E). Similarly, in the case of downregulated DEGs, genes such as *KRT34* and *GPRIN2* show expression in OSF without dysplasia only, and genes such as *SLC6A17* and *CYP4Z2P* show expression only in OSF with dysplasia (Figure 6F).

We further performed ROC analysis and plotted AUC curves using the GSE64216 dataset to evaluate how well each single gene can discriminate between OSF and normal samples. In the case of upregulated DEGs, 22 individual AUC curves were plotted, out of which 13 genes discriminate OSF patients from normal with the correct direction (higher in OSF). Out of these 13 genes, six genes *(CYP24A1, DHCR7, IGF2BP2, MDFI, PI15,* and *SERPINE1*) show an AUC of 0.94 and above, and the remaining seven genes show an AUC of 0.54 and above. The remaining nine upregulated DEGs show higher expression in normal instead of OSF. Likewise, we plotted AUC curves for 11 downregulated DEGs and observed 7/11 genes showing a positive trend, i.e., higher in normal, and the remaining four genes (*SLC6A17, ECM1, CYP4Z2P,* and *KRT34*) showing the opposite trend, i.e., higher in OSF. Out of seven genes, four genes show AUC >0.90 (Figure), and three genes show AUC >0.60. AUC plots for upregulated and downregulated DEGs with AUC >0.90 are shown in Figures 6G–P, respectively, and the remaining figures for upregulated and downregulated DEGs are shown in Supplementary Figures S3, S4, respectively.

### 3.7 Epigenetic analysis reveals hyper and hypo methylated genes

**FIGURE 7:**
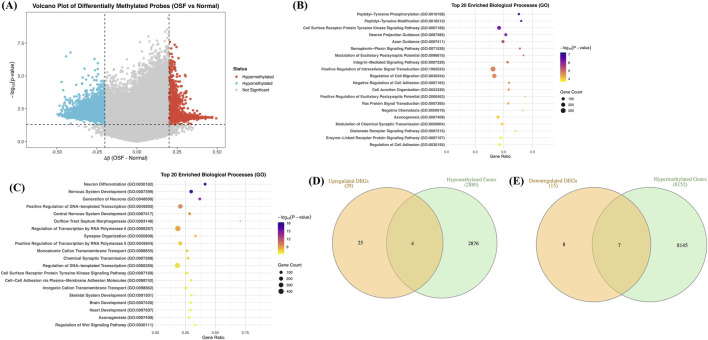
Epigenetic analysis complements transcriptomic analysis. Panel **(A)** represents the violin plot of differentially methylated probes (OSF vs. normal) along with delta beta values on the x-axis and -log10 (p-value) values on the y-axis. Panel **(B,C)** represent the top 20 enriched biological processes associated with hyper- and hypomethylated genes, respectively. The Venn diagram in panel **(D)** shows the common genes between upregulated DEGs and hypomethylated genes, whereas panel **(E)** shows the common genes between downregulated DEGs and hypermethylated genes.

We were able to obtain one dataset, GSE156669 by Kundu et al., where they looked at the genome-wide methylation pattern in OSF patients and normal people. For each CpG site, beta methylation values for both OSF and normal patients were analyzed. We looked at the delta beta value, i.e., the average beta value in OSF patients minus the average beta value in the normal population, and observed the range varied between 0.5 and −0.5 (Figure 7A). Positive delta beta values represented hypermethylation, and negative values represented hypomethylation. Next, we obtained the list of genes showing positive and negative delta beta values ≥0.1 and ≤ −0.1, respectively. In total, we obtained a total of 8152 unique hypermethylated genes and 2880 hypomethylated genes (Supplementary Table S16).

We looked at the enriched biological processes associated with these genes and found that hypermethylated genes are associated with processes such as “Peptidyl-Tyrosine Phosphorylation,” “Integrin-mediated Signaling Pathway,” “Regulation of Cell Migration,” etc., highlighting the epigenetic silencing of key regulators of intracellular signaling and fibroblast-ECM communications (Figure 7B). Hypermethylation of the genes involved in pathways such as integrin and tyrosine kinase could dysregulate normal feedback control, sustaining activation of fibroblasts and excessive deposition of collagen (Moretti et al., 2022). In addition, alterations due to methylation in genes related to migration may impair tissue remodeling and promote fibrotic persistence (Zhang X et al., 2017). These observations collectively reflect the fact that aberrant DNA methylation leads to dysregulated signaling and fibrogenesis in OSF. List of complete processes associated with hypermethylated genes is provided in Supplementary Table S17.

Likewise, we looked at the enriched processes associated with the hypomethylated genes and observed processes such as “Neuron differentiation,” “Transcriptional regulation,” “Cell-cell adhesion,” etc., suggesting activation of the developmental and signaling processes that are normally not active in normal oral mucosa (Figure 7C). Furthermore, activation of neuronal pathways may link to neuropathic pain and altered innervation, a key characteristic of OSF (Kocot-Kępska et al., 2021). Similarly, upregulation of transcriptional regulators and tyrosine kinase signaling components may enhance cell proliferation and survival, supporting disease progression and malignant transformation into OSCC (Zhang N, and Li, 2023). These observations reflect that hypomethylation of genes in OSF promotes ectopic activation of developmental and signaling networks, resulting in both fibrotic and pre-neoplastic changes (Tan et al., 2023). List of complete processes associated with hypomethylated genes is provided in Supplementary Table S18.

Next, we compared the list of hypomethylated genes with our upregulated DEGs and hypermethylated genes with downregulated DEGs (Figures 7D,E), as genes with lesser methylation in promoter regions are usually more expressed and genes with higher methylation in promoter regions are usually less expressed. As a result, we observed four common genes, *CYP24A1, FBLIM1, LY6K,* and *MDFI*, showing hypomethylation and upregulation, and seven genes, *CXCR2, CYP34A, ECM1, ERC2, GPRIN2, SLC6A17,* and *ST6GAL2.*

The above integrative transcriptome-methylome analyses show their potential as diagnostic and prognostic markers. Upregulation of *CYP24A1, FBLIM1, LY6K,* and *MDFI* is largely associated with ECM remodeling, fibroblast proliferation, and driving malignant transformation, a characteristic feature of OSF. Conversely, downregulation of *CXCR2, CYP34A, ECM1, ERC2, GPRIN2, SLC6A17,* and *ST6GAL2* is largely associated with regulator or protective functions such as preventing inflammation, ECM turnover, or differentiation. Hence, consistent expression and methylation of these genes can be used for early diagnosis, and the progressive changes in their expression or methylation could predict the risk of malignant transformation.

### 3.8 Single cell analyses show higher expression of DEGs in key cell types

**FIGURE 8:**
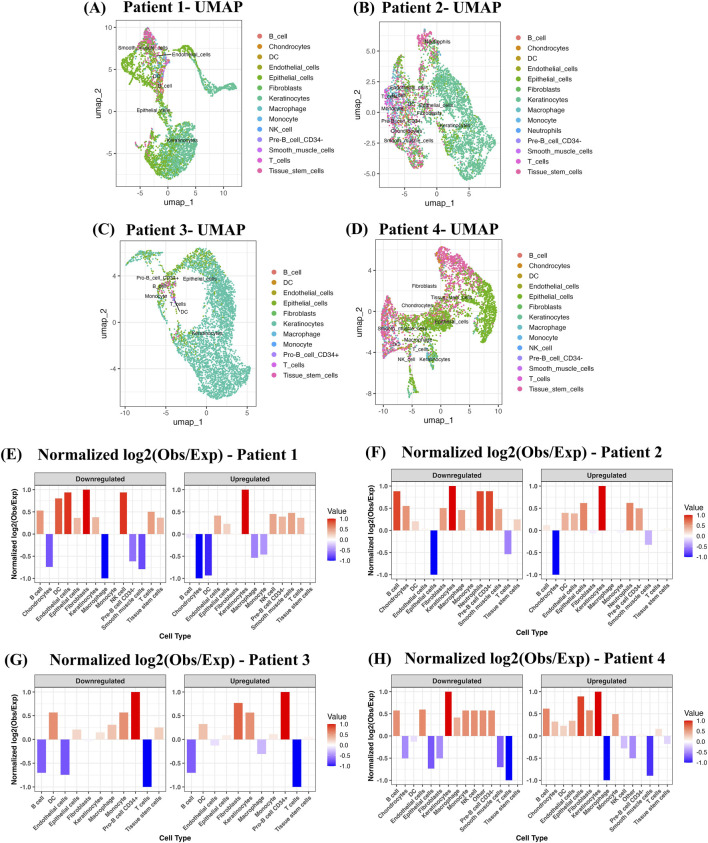
Single-cell analysis reveals involvement of key cell types in OSF. Panel **(A–D)** shows the UMAP visualization of single-cell transcriptome annotated using the package SingleR. Panel **(E–H)** shows the gene expression pattern of the up- and downregulated DEGs in each cell type. For each gene across cell type, the average z-score was computed in that cell type, followed by computing the log2 (Obs/Exp) fraction of genes expressed in each cell type and further normalizing the values between −1 and 1.

We downloaded the raw data from the GEO and processed it using the standard Seurat v.5 pipeline. Quality control was performed to remove the low-quality cells and mitochondrial reads (see Methods). Next, we annotate the cell types using the Human Primary Cell Atlas and the SingleR package using default parameters. The analysis was performed for each of the four patients, and we observed enrichment of various cell types in each patient, where most of the cell types were common and some were unique. For example, cell types such as fibroblasts, keratinocytes, and epithelial cells were enriched among all the patients, highlighting the role of key immune processes during the infection. We also observed other key cell types such as pre-B cell CD34^−^, smooth muscle cells, tissue stem cells, *etc.*, Suggesting dynamic microenvironment collectively contribute to tumor progression in OSF-driven oral cancer. For example, presence of pre-B cell CD34^−^, reflect localized adaptive immune infiltration due to chronic inflammation in OSF. Likeiwse, smooth muscle or myofibroblast-like cells indicates stromal remodeling and ECM stiffening driven by fibrotic tissues, while tissue stem cells point towards regenerative pressure and emergence of stem-like populations leading to tumor initiation and progression. “Neutrophils” were observed only in patient 2, indicating selective activation of the immune response or inflammation (Figures 8A–D).

Next, we looked at the expression of DEGs in each individual cell type to observe the expression pattern. We computed the z-score for each gene across cell type and then computed the average z-score for each gene in that cell type. After this we computed the Log2 (Obs/Exp) fraction of genes expressed in each cell type and further normalized the values between −1 and 1 (see Methods). As shown in Figures 8E–H, we observed that upregulated DEGs show high expression in the “Keratinocyte” cell type, followed by “Epithelial cells” in all four patients. This finding indicated the involvement of the epithelial transcriptional program in OSF-driven OSCC. It underscores the pivotal role of interaction between epithelial-mesenchymal cells, keratinocyte activation, and remodeling of the barrier in driving fibrosis and OSF tissue pathology characteristics. Upregulated DEGs also show high expression in cell types such as “fibroblast”, “endothelial” and “pre-B cell CD34-” in some cases.

In the case of downregulated DEGs, we observed “dendritic cells” and “keratinocytes” to be enriched among all four patients. This observation shows the coordinated suppression of immune function and epithelial differentiation programs as antigen presentation activity via DC cells and keratinocyte function is getting reduced, which together may lead to an immunosuppressive and fibrotic microenvironment. We also observed enrichment of other cell types such as “B cells,” “monocytes,” “macrophage,” *etc.*, in some of the patients. This observation also shows the heterogeneity of gene expression in individual patients and how they regulate OSF pathology.

## 4 Discussion

Oral submucosal fibrosis (OSF) is chronic, progressive, and irreversible in nature, which is caused mainly due to areca nut chewing, particularly in South Asian countries. OSF is characterized as a disease with a malignant tendency; however, the specific pathogenesis of its origin and malignant transformation is not clear yet. Despite enormous efforts, non-invasive, effective diagnostic approaches and strategies for precision therapy and prevention are the goal to eliminate this disease. In the current study, we have performed an integrative multi-omics approach to find new targets that can be used as novel diagnostic and prognostic biomarkers and therapeutic targets.

Although our current work uses a systems-level approach that incorporates transcriptomics, epigenomics, and single-cell data, the true strength of such approaches lies in their capacity to address the complexity of regulation at different levels of biology. For example, transcriptomics allows for the analysis of regulation at the expression level, epigenomics allows for a detailed analysis of regulation at a chromatin or regulatory level, and single-cell analysis allows for a detailed analysis of heterogeneity that may not be apparent at a population or tissue level. By integrating all these different data modalities, a more comprehensive level of understanding of disease biology can be achieved. A review by Ubaid et al. has very nicely highlighted how multi-omics data integration can help in identifying oncogenic determinants (driver genes, regulatory networks, etc.) across different cancers (Ubaid et al., 2025).

Here, our work extends this multi-omics integration principle in the context of OSF, a pre-malignant condition. It is not only a disease-specific study but also a means of understanding the early pathogenic events and microenvironmental reprogramming associated with fibrosis and cancer. By integrating RNA-seq (bulk and single-cell) with epigenomic regulation, we move beyond simple characterization of the disease process and towards a mechanistic and systems-level approach, thereby improving the opportunity for identifying clinically relevant biomarkers and therapeutic targets.

First, we analyzed several RNA-seq datasets specific to OSF and characterized DEGs (up- and downregulated) and performed several analyses. Gene enrichment analyses revealed key processes associated with DEGs. In the case of upregulated genes, we observed enrichment of terms associated with inflammatory, metabolic, and signaling pathways, which are associated with chronic inflammation and disease progression. For example, TNF-α and IL-1β promote activation of fibroblasts and excessive collagen deposition, a key characteristic feature of OSF (Rajalalitha, and Vali, 2005). Likewise, downregulated genes were largely associated with metabolic processes, which play a key role in disease progression. For example, dysregulation in metabolic balance affects antioxidant defense and epithelial integrity, ultimately leading to fibrosis. Along with biological processes, we also looked at the enriched molecular functions and cellular compartments linked to DEGs. KEGG pathways associated with upregulated genes include steroid biosynthesis, Hippo signaling, etc., where any abnormality in the functioning of these pathways will lead to collagen accumulation and fibrotic events (El-Okdi et al., 2008), whereas downregulated genes were associated with metabolic pathways such as linoleic acid metabolism, N-Glycan biosynthesis, etc. Additionally, disease terms such as “keloid,” “fibroid tumor,” *etc.*, Were linked with upregulated DEGs, and terms such as “physical aggression,” “myeloid metaplasia,” *etc.*, Were associated with downregulated DEGs.

Protein-protein interaction analysis shows interaction between upregulated DEGs; however, no interaction was observed for downregulated DEGs. We further complemented the sparse interaction observed in PPI analysis by performing pathway-level activity assessment using GSVA analysis to evaluate the functional consequences of the identified gene interactions. Our analysis showed increased activity of key pathways associated with OSF pathogenesis, such as TGF-β signaling, TNFα signaling via NF-κB, IL6-JAK-STAT3 signaling, complement activation, and epithelial-mesenchymal transition in treated samples.

Immune cell enrichment analysis using the CIBERSORTx and xCell shows higher enrichment of T cells (CD4 memory resting, CD8), mast cells, and macrophages (M1 and M2) in OSF patients, reflecting persistent antigenic stimulation and forming an immune microenvironment consistent with chronic inflammation, potential immune exhaustion, and fibrotic events (Galli, and Tsai, 2008). In contrast, dendritic cells and natural killer cells were more enriched in normal patients. Further, Spearman correlation among 22 immune cell types shows higher positive correlation of M2 macrophages with monocytes and T cells CD8 and of M1 macrophages with T cells CD8 and T cells gamma delta. This correlation highlighted the coordinated activation of the innate immune system, highlighting their shared origin from myeloid cells and role in tissue remodeling and inflammation. In addition, we also observed strong negative correlations between resting and memory B cells, NK cells, and subsets of T cells (T cells CD4) with monocytes, macrophages, etc., highlighting a reciprocal balance between the innate and adaptive immune systems, a well-established phenomenon in diseased conditions such as cancer and fibrosis.

Next, we validated our DEGs (up- and downregulated) in an independent cohort of two microarray transcriptomic datasets, where we found data for 22 common upregulated and 11 downregulated DEGs. Firstly, we developed several ML models using the training data in five-fold cross-validation settings. Among all models, Adaboost classifier perform best with AUROC of 0.96 and AUPRC of 0.97. We further used RFE technique for selecting top5 features for developing models, where Random Forest model performed best and achieved AUROC of 0.99 and AUPRC of 0.99. Secondly, we looked at the gene expression pattern in the new dataset, we found that most of our DEGs show the correct trend of gene expression, i.e., upregulated DEGs show higher expression and downregulated DEGs show lower expression in OSF patients. We also observed a pattern within OSF patients with dysplasia and without dysplasia as well when we plotted the gene expression in the case of dataset GSE64216. Next, we performed ROC analysis and plotted AUC curves and observed 13/22 upregulated DEGs can discriminate OSF patients from normal counterparts with high AUROC, and 7/11 downregulated DEGs can discriminate OSF patients from normal counterparts with high AUROC. Furthermore, nested cross-validation technique followed by permutation testing further supported the model performance.

We also analyzed the methylation dataset and selected the hypermethylated and hypomethylated genes and looked at their enriched terms. Hypermethylated genes were largely associated with processes related to signaling and cell migration pathways, whereas hypomethylated genes were found to be associated with processes such as neuron differentiation, transcriptional regulation, cell-cell adhesion, etc. Afterwards, we looked for the common genes that show higher gene expression and hypomethylation and found four key genes, satisfying the criteria. Likewise, we analyzed genes, which showed lower gene expression and hypermethylation, and found seven key genes, fulfilling the criteria. These integrative transcriptome-methylome analyses show their potential as diagnostic and prognostic markers, as consistent expression and methylation of these genes can be used for early diagnosis, and the progressive changes in their expression or methylation could predict the risk of malignant transformation.

Lastly, we analyzed the single-cell dataset to look for the cell types enriched in OSF patients compared to normal. First, we annotated the cell types using the SingleR package and saw enrichment of common and unique cell types in patients. Next, we analyzed the DEGs expression in each cell type by computing the Log2 (Obs/Exp) z-score and observed that upregulated DEGs show higher expression majorly in keratinocyte and epithelial cell types, whereas downregulated DEGs show higher expression majorly in keratinocyte and dendritic cell types. This observation underscores the crucial role of interaction between epithelial-mesenchymal cells, keratinocyte activation, and remodeling of the barrier in driving fibrosis and OSF tissue pathology characteristics.

## 5 Conclusion

In conclusion, our study characterizes a novel gene signature that can be used as a potential diagnostic biomarker and therapeutic target. We characterize DEGs from the RNA-seq datasets and perform several analyses. Gene enrichment analyses revealed OSF-specific processes, pathways, and disease terms. PPI analysis shows interaction among upregulated genes, and immune cell infiltration analyses provided a list of key immune cell types enriched in OSF patients, such as T cells, mast cells, and macrophages. Further, we validated our initial findings in independent datasets where we looked at the gene expression pattern and direction. ROC and AUC plots show that DEGs can discriminate OSF patients from the normal population with high accuracy. Epigenomic analyses identified key hypermethylated and hypomethylated genes. Integrative analyses of epigenetic and transcriptomic datasets provided 11 gene signatures, which can act as potential novel diagnostic biomarkers and can also be used as therapeutic targets. Single-cell analyses highlighted the fact that DEGs show higher expression in cell types such as keratinocytes, epithelial cells, and dendritic cells. Our study provides the mechanistic insight into the OSF pathobiology and potential mechanism leading to oral cancer.

## Data Availability

The original contributions presented in the study are included in the article/Supplementary Material, further inquiries can be directed to the corresponding author.
