# Supplementary material for: Delineating novel diagnostic biomarkers and therapeutic targets for oral submucosal fibrosis: an integrative multi-omics and machine learning approach
Source: Front Bioinform. 2026 May 4;6:1803111. doi: 10.3389/fbinf.2026.1803111 (PMC13180744; doi:10.3389/fbinf.2026.1803111)
Supplement: Supplementary file 1 [file Supplementaryfile1.docx]

**Delineating novel diagnostic biomarkers and therapeutic targets for oral submucosal fibrosis: An integrative multi-omics and machine learning approach**

Chinmay Nitin.Mokal^1^ and Piyush Agrawal*^2^

1. Department of oral and maxillofacial surgery, SRM Kattankulathur Dental College & Hospital

2. Division of Medical Research, SRM Medical College Hospital, SRM Institute of Science and Technology, Kattankulathur, Chennai, India

**Corresponding author**

Piyush Agrawal, Ph.D.

Division of Medical Research, Research Centre, SRM Medical College Hospital, SRM Institute of Science and Technology, Kattankulathur, Chennai, India

**Email:** [piyusha@srmist.edu.in](mailto:piyusha@srmist.edu.in), [apiyush74@gmail.com](mailto:apiyush74@gmail.com)

**ORCID:** 0000-0003-2075-1111

(A)

(B)

**Supplementary Figure S1. Molecular Function Analysis**. Panel (A) shows top20 enriched molecular functions associated with upregulated DEGs, whereas Panel (B) shows top20 enriched molecular functions associated with downregulated DEGs.

(A)

(B)

**Supplementary Figure S2. Cellular Compartment Analysis**. Panel (A) shows enriched cellular compartments associated with upregulated DEGs, whereas Panel (B) shows enriched cellular compartments associated with downregulated DEGs.

**Supplementary Figure S3. ROC analysis and AUC plot.** Panel (A-P) shows the ROC analysis and AUC plot for the upregulated DEGs discriminating OSF patients from normal.

**Supplementary Figure S4. ROC analysis and AUC plot.** Panel (A-G) shows the ROC analysis and AUC plot for the downregulated DEGs discriminating OSF patients from normal.


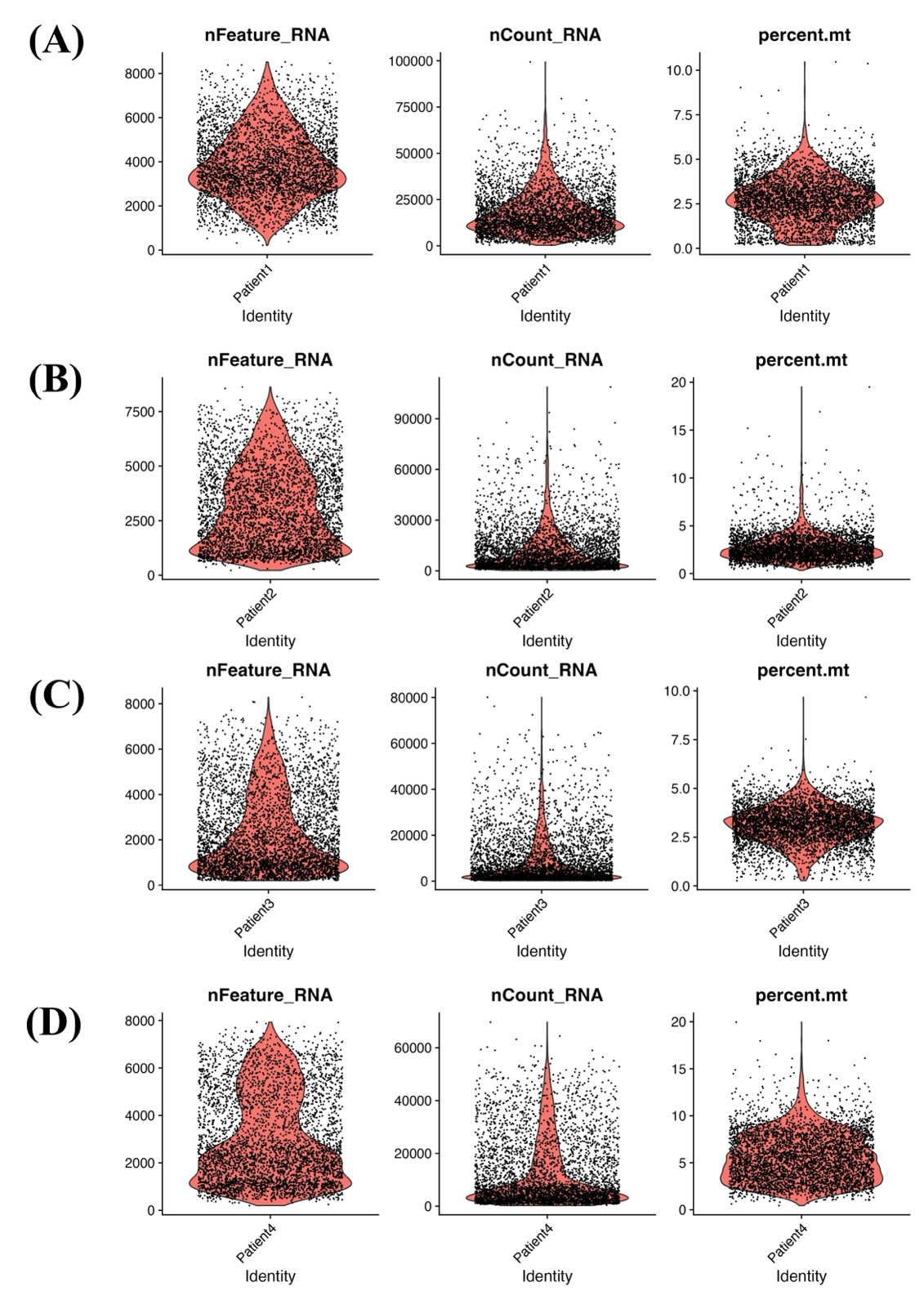


**Supplementary Figure S5.** **Quality control analysis for spatial transcriptomic data.** The violin plots demonstrating quality control metrics for each individual patient sample, in terms of number of detected genes per cell (nFeature_RNA), total transcript count (nCount_RNA), and percent of mitochondrial expression (percent.mt). Low-quality or damaged cells were filtered out, and only high-quality cells were retained, as indicated by those with low feature count and high mitochondrial content.
